# Pulse generation with ultra-superluminal pulse propagation in semiconductor heterostructures by superradiant-phase transition enhanced by transient coherent population gratings

**DOI:** 10.1038/lsa.2016.86

**Published:** 2016-06-03

**Authors:** Peter P Vasil'ev, Richard V Penty, Ian H White

**Affiliations:** 1 Centre for Photonic Systems, Department of Engineering, University of Cambridge, Cambridge CB3 0FA, UK; 2 Quantum Electronics Division, PN Lebedev Physical Institute, Moscow 119991, Russia

**Keywords:** phase transition, population grating, superradiance, superluminal propagation

## Abstract

This paper reports the observation of ultra-superluminal pulse propagation in multiple-contact semiconductor heterostructures in a superradiant emission regime, and shows definitively that it is a different class of emission from conventional spontaneous or stimulated emission. Coherent population gratings induced in the semiconductor medium under strong electrical pumping have been shown to cause a major decrease of the group refractive index, in the range of 5–40%. This decrease is much greater than that caused by conventional carrier depletion or chirp mechanisms. The decrease in refractive index in turn causes faster-than-c propagation of femtosecond pulses. The measurement also proves the existence of coherent amplification of electromagnetic pulses in semiconductors at room temperature, the coherence being strongly enhanced by interactions of the light with coherent transient gratings locked to carrier gratings. This pulse-generation technique is anticipated to have great potential in applications where highly coherent femtosecond optical pulses must be generated on demand.

## Introduction

The study of cooperative emission from an ensemble of quantum oscillators, often referred to as superradiance (SR) or superfluorescence, was triggered by a pioneering paper by Dicke in 1954^1^. Since then, exciting theoretical and experimental research has focused on the collective quantum behavior and SR emission from different types of media, including gases, solids, polymers, Bose condensates, and 0 dimensional (D), 2D and 3D semiconductors^2, 3, 4, 5, 6, 7, 8^. The model describing the interaction of matter with electromagnetic radiation proposed by Dicke has proven to be of key importance for studying the collective, coherent and dynamic effects in quantum optics^9^. In practice, the real interaction between electromagnetic emission and matter is too complicated for a complete theoretical investigation. However, Dicke devised a significant simplification, enabling him to consider these physical phenomena within the framework of a simple analytic model that has precise solutions. He assumed that the system is composed of *N* emitters cooperatively interacting with a single radiation field mode. The essence of cooperative behavior is that the oscillator dipoles interact coherently with the privileged radiation mode. One of the most striking phenomena provided by the Dicke model is the ability of the system to exhibit a second-order phase transition from a normal to a superradiant state at a certain critical temperature^10, 11, 12^. In recent years, the Dicke model has drawn renewed interest because it is a simple system in which one can find entanglement and related phenomena, and because it can be realized in a wider range of systems than in the original cavity quantum electrodynamics case. A new aspect emerged when it was realized that the SR quantum-phase transition is relevant to quantum information and quantum computing^13^.

Among the most fascinating and important phenomena that occur during an SR-phase transition are the self-organization of emitters and the formation of a macroscopically ordered state. The mutual phasing of emitters involved in radiative emission, their self-organization and self-order originate from an exchange of photons of an internal electromagnetic field. Strong pumping and large field-matter coupling are the crucial factors that lead to a discontinuous change of the system from a normal to a superradiant state. For example, the experimental observations of the Dicke quantum-phase transition in Bose condensates of ultracold atoms^14^ and microcavity polaritons at cryogenic temperatures^15^ require high-finesse optical cavities and strong pumping of the system above the critical level. The self-organization of emitters results in regular 1D (grating-like) or 2D (chequerboard-like) spatial distributions of the inverted populations. Likewise, two counter-propagating SR pulses burn a periodic modulation or ‘grating’ into the inverted population distributions, the period of the modulation being half the optical wavelength. This lattice or grating results in a backscattering and pulse correlation^16, 17^. A distinctive feature of population gratings in semiconductor materials is that they result in strong refractive index gratings, which result in a change in the group velocity of light. This has recently attracted considerable interest within the physics community^18, 19, 20, 21^, not least because the control of the speed of the propagation of light could potentially find a number of applications in optical communications, photonics, optical storage and other fields of quantum electronics.

Various papers have reported SR phenomena in semiconductors at room temperature^22, 23, 24, 25^. However, such observations have led to debate on whether the pulses generated are really akin to those superradiant phenomena observed in low temperature or low-density structures^5, 6, 7^ and whether they are different, for example, from those generated by conventional Q-switching^26^. In this work, by measuring the superluminal propagation properties of the pulses generated in GaAs/AlGaAs multiple-contact heterostructures operating in a superradiant emission regime, we demonstrate for the first time the existence of transient coherent gratings directly coupled to the amplified pulses. In this paper we use ‘superluminal’ to mean that SR pulses travel in the medium substantially faster than any pulses during normal lasing or spontaneous emission in the same medium. We describe a decrease of the group refractive index in the range of 5–40%, resulting in strong superluminal behavior not observed in conventional spontaneous or stimulated processes and due to the SR-phase transition. We present experimental and theoretical results of the superluminal pulse propagation during the SR-phase transition in the semiconductor medium at room temperature due to coherent transient refractive index gratings.

## Materials and methods

### Experiment

**Figure 1:**
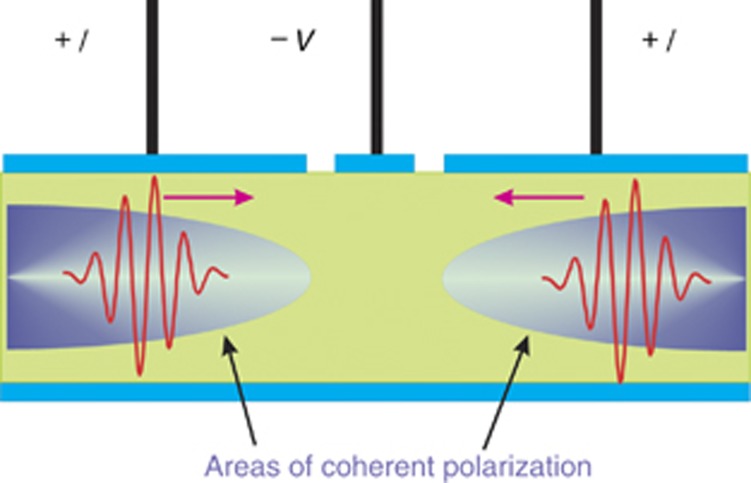
Schematic of the semiconductor structure. The forward driving current *I* and the reverse bias *V* are applied to the end and central sections, respectively.

A wide variety of multiple-section GaAs/AlGaAs bulk laser structures capable of generating SR emission have been studied. In general, they have gain and saturable absorber sections of different geometries with different gain/absorber ratios and total cavity lengths. The devices under test are described in detail in our previous publications (see, for instance, refs. 22, 23, 24, 25). The varied composition of the GaAs/AlGaAs heterostructures results in a broad range of operating wavelengths (between 820 and 890 nm). All devices can operate under continuous wave operation, gain/Q-switching or SR regimes, depending on the driving conditions^26^. A typical 3-section laser structure is schematically illustrated in Figure 1. The two end sections are pumped by nanosecond current pulses, providing two areas with high e–h density. The center section is reverse-biased and is a controllable, saturable absorber. Generated pulses can travel back and forth between the chip facets, whose power reflectivity coefficients are ~0.32. Regions of coherent polarized amplification (the macroscopically ordered state of the e–h system) exist at both ends of the structure during the superradiant-phase transition because these are the regions that are strongly pumped^22, 23^. The exact values of the cavity lengths of devices are measured using scanning electron microscopy with an accuracy of better than 1 μm because accurate values are required for the calculation of the group refractive index.

We calculate variations of the group refractive index using measurements of both the longitudinal mode spectral spacings and the round-trip times (RTTs) of pulses in different dynamic regimes. Optical emission spectra are analyzed using an optical spectrum analyzer with a spectral resolution of 0.07 nm. The RTT is measured with femtosecond accuracy by a fringe-resolved autocorrelation technique based on second harmonic generation (SHG)^26^ or using a single-shot streak camera with an ultimate temporal resolution of ~1.5 ps.

**Figure 2:**
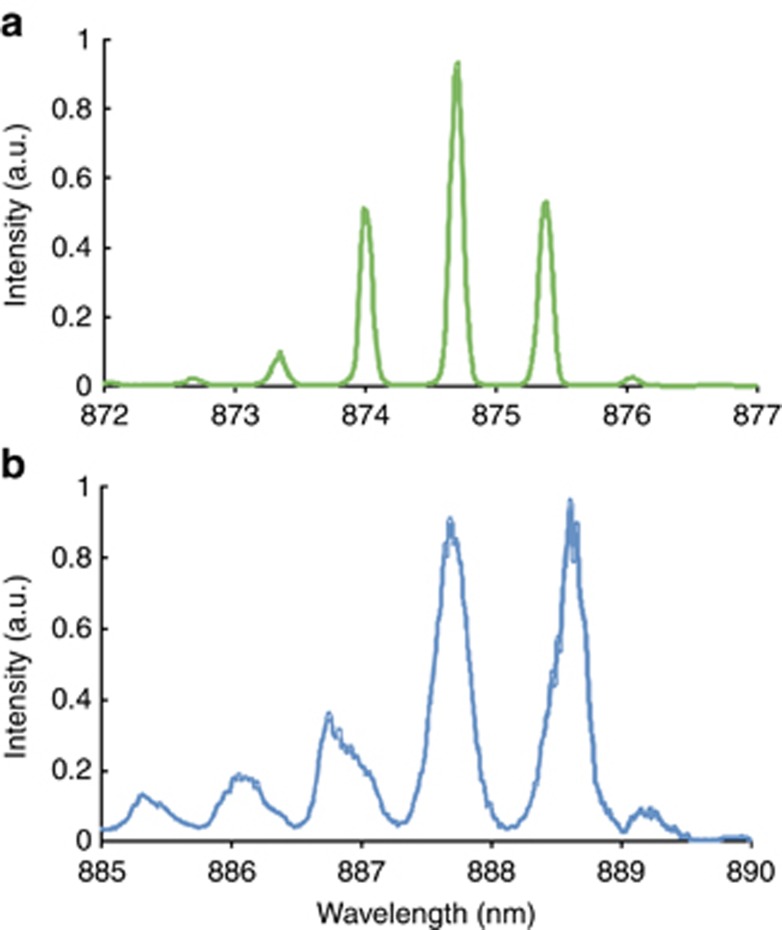
(**a**) ‘-’ c.w. lasing emission spectrum of a 150-μm-long, 3-section structure at *I*=132 mA and *V*=0.0 V. (**b**) ‘–’ SR optical spectrum at *I*=380 mA, *V*=−4.4 V. Note the red shift of the SR spectrum.

Figure 2 presents typical optical spectra of standard continuous wave (c.w.) lasing (a) and SR emission (b) generated from the same 3-section structure at different reverse bias levels and gain currents. SR spectra are always red shifted by 10–20 nm with respect to the peak of lasing because the observed cooperative emission occurs at the band-gap energy^22, 23, 24, 25^. The peak wavelength of the SR emission is ~889 nm, whereas the center wavelength of the c.w. emission is located at 875 nm. The measured-mode spacing in Figure 2a and 2b is 0.67 and 0.93 nm, respectively. As expected, the measured-mode spacing of the lasing spectra is dependent on the cavity length but does not depend on the driving conditions. By contrast, SR spectra exhibit a clear dependence of the spacing on current and voltage.

**Figure 3:**
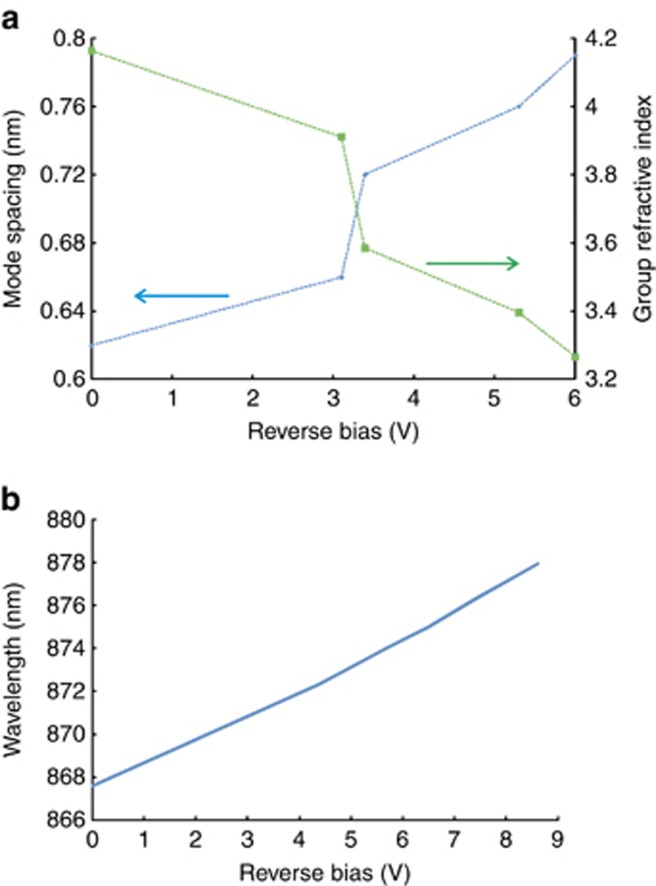
Dependence of the SR mode spacing and the corresponding change of the group refraction index (**a**) and peak wavelength (**b**) on the reverse bias *V*.

We have experimentally observed that samples with different cavity lengths exhibit different levels of decrease in refractive index. Figure 3 presents the experimentally measured changes to the mode spacing and the group refractive index with increasing voltage *V* to the absorber section of another sample. The group refractive index is calculated according to the relation *n*_*g*_(*λ*)=*λ*^2^/2*L*Δ*λ*(*λ*), where *λ* is the central wavelength, *L* is the chip length and Δ*λ* is the mode spacing, which depends on *λ* due to material and waveguide dispersion.

**Figure 4:**
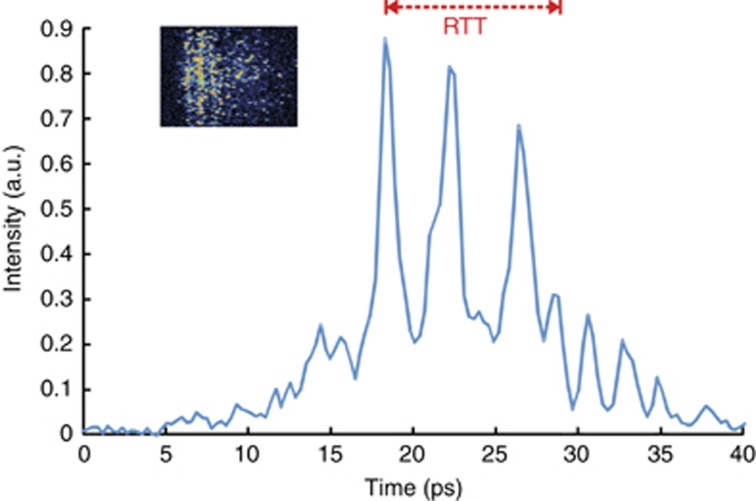
Photo from the streak camera screen and corresponding SR pulses. The RTT of the cavity related to lasing is shown by the red arrow.

Figure 4 shows a typical burst of SR pulses from a device 450 μm long. There are two pulses on the period of the resonator. The number of pulses is normally between two and six, depending on driving and the cavity length. The pulse widths of the individual pulses are <1 ps, and are accurately measured using the SHG autocorrelation technique^26^. The common feature of all SR pulses is that the separation between the pulses is always smaller than a half of RTT, which is measured for the standard lasing regime in the same semiconductor structure.

**Figure 5:**
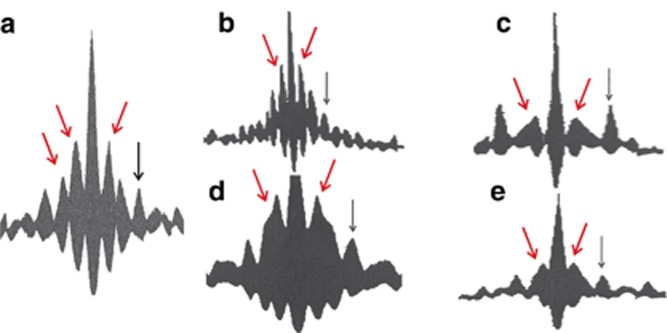
Autocorrelation traces of SR pulses at different values of *I* and *V*: 730 mA and −6.9 V (**a**); 730 mA and −7.4 V (**b**); 464 mA and −4.3 V (**c**); 677 mA and −5.1 V (**d**); 383 mA and −3.9 V (**e**). The device length is 98 μm, which corresponds to a RTT of ~3.1 ps. The absorber length is 30 μm. The peaks at RTT are marked by the black arrows; the additional peaks caused by the population gratings are shown by red arrows.

Figure 5 shows a number of typical fringe-resolved autocorrelation traces of SR pulses. The detection of SHG autocorrelation traces allows for the direct and accurate measurement of the RTT. A fringe-resolved autocorrelation trace of any laser emission exhibits a *single* peak at zero delay^26^, and its width determines the coherence time of the laser emission and is inversely proportional to the spectral bandwidth. For mode-locked pulses, there are additional *identical* peaks separated by the RTT of the laser cavity. The autocorrelation traces in Figure 5a–5e measured for SR pulses show additional structures or oscillations in addition to the structures of lasing. This can be explained by assuming that there is an oscillatory process linking the electromagnetic field and the e–h system typical of grating locking. The shape and number of coherent oscillations in Figure 5a–5e depend strongly on the driving conditions (both *I* and *V*) and the geometry of the gain/absorber sections, unlike those for lasing, which do not change significantly. SR emission travels along the longitudinal axis between the chip facets, successfully being reabsorbed and re-emitted in a similar way to Rabi-type oscillations. Multiple peaks and fringes at nonzero delays originate from the coherent interaction of the electromagnetic field with carrier density transient gratings. These peaks are marked by the red arrows and, as discussed in theoretical modeling section below, they never exist without the coherent population gratings.

**Figure 6:**
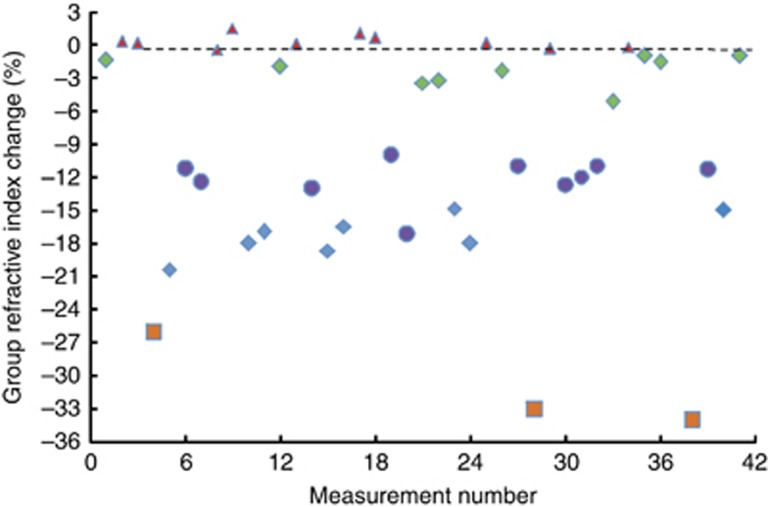
Measured variations of *n*_*g*_ for different devices and different driving conditions. The initial values of the refractive index were calculated for lasing near the threshold. ▴: Q-switching; 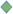: SR, 450 μm cavity; 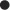: SR, 350 μm; 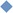: SR, 150 μm; ▪: SR, 100 μm.

The measurement of RTTs under lasing has been conducted to demonstrate the independence of these times on the driving conditions. A large number of measurements of over 20 devices of different lengths (from ~100 to 450 μm) were performed, as shown in Figure 6. The change of the group refractive index for the Q-switching with respect to c.w. laser emission is <2%, whereas it is typically within 3–23% in case of SR, the largest measured values being ~35%. We observe a correlation between a decrease of *n*_*g*_ and the length of the structure, with the maximum decrease being for the shortest devices. The measurement conditions in Figure 6 are the same for all samples. The driving current and the reverse voltage for each device and each measurement are adjusted to achieve the shortest and highest power SR pulses. The driving conditions (the value of *I* and *V*) for achieving this criterion for the samples of different lengths are different.

### Theoretical modeling

The physical reason behind the ‘fast light’ phenomenon during the SR-phase transition appears to be different from the ‘fast/slow light’ effects that have been recently observed in resonant media with population inversion^18, 19, 20, 21^. Indeed, the semiconductor-active medium in the GaAs/AlGaAs heterostructures does not exhibit closely spaced gain or absorption lines with a strong anomalous dispersion region that can be responsible for the fast light effect. However, in contrast to a laser, where the field is coherent and the active medium is incoherent, macroscopically large areas of the coherent macroscopic population exist in the semiconductor during the SR-phase transition^22, 23, 24, 25^. It has previously been suggested that superluminal propagation of light in a coherent amplifier can occur as a result of pulse reshaping^27, 28, 29, 30, 31^.

The coherence of the semiconductor medium, as well as the induced coherent population gratings, plays a decisive role in the phenomenon under study. Indeed, reflections of the electromagnetic field at the chip facets lead to the co-existence of two counter-propagating SR pulses. Due to the coherence of the e–h system, these pulses each form a coherent transient grating with a spatial period equal to a half of the wave vector of the light *k*. Nonlinear interaction of the SR pulses with coherent gratings can result in variations of their group velocity. The impact of this mechanism on superluminal pulse propagation can be clarified by solving the Maxwell–Bloch equations^9, 32^, with the explicit inclusion of the spatial carrier grating. The spatial distribution of the carrier density can be expressed as follows^9, 33^:


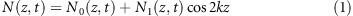


The system of equations, which is governed by the dynamics of the SR pulse generation, is given in dimensionless variables as^9, 17, 32^


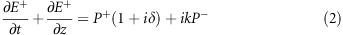



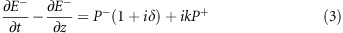



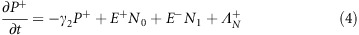



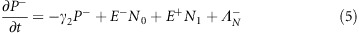



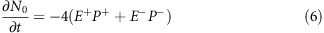



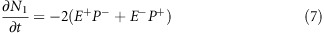


where *E*^*±*^ and *P*^*±*^ are the slowly varying amplitudes of the counter-propagating electric fields and the active medium polarizations, respectively, and *γ*_2_ is the dimensionless polarization relaxation rate. As we are interested in ultrafast phenomena occurring on a time scale of a few picoseconds, we neglect the terms describing the drive current, spontaneous emission and carrier diffusion. Equations (2) and (3) explicitly include the terms responsible for the interaction of the fields with the carrier grating, as in the modeling of colliding pulse mode-locked semiconductor lasers, where the carrier grating in the absorber plays a decisive role^26, 34^. The strength of the interaction is determined by the coefficient *κ*, which is proportional to *N*_1_^34^. In contrast to SR generation by solid-state media and gases, there is a strong relation between the refractive index and the carrier density in semiconductors. The parameter *δ* takes into account changes in the refractive index due to variations of *N*. *δ* is proportional to the linewidth enhancement factor^34^. Equations (6) and (7) include *δ*-correlated Gaussian random functions Λ^*±*^_*N*_ describing spontaneous noise in a manner similar to that in our previous SR model^25^. Equations (2), (3), (4), (5), (6), (7) were solved with the initial and boundary conditions and the material parameters used in our previous SR models for the experimental samples^35, 36^.

**Figure 7:**
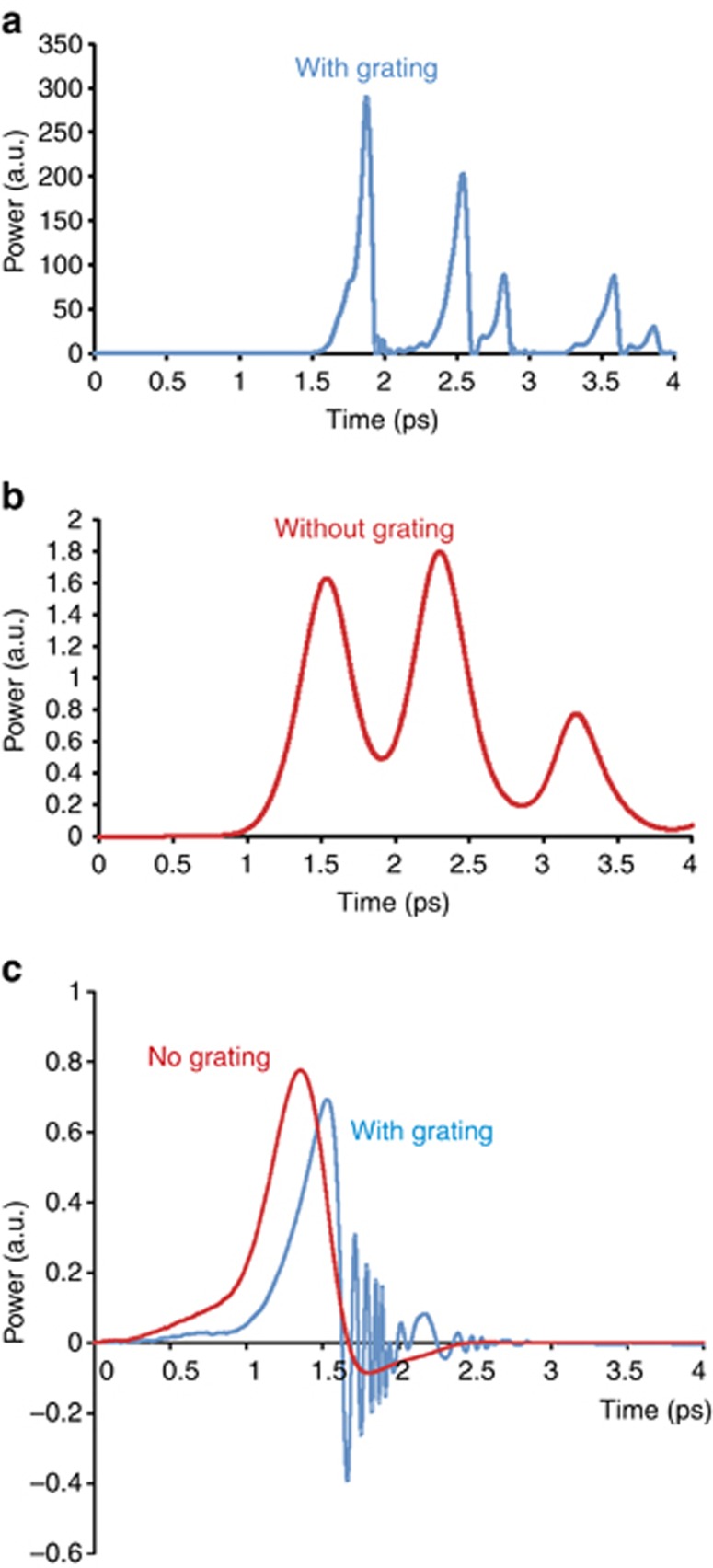
Calculated SR pulses with (**a**) and without (**b**) the grating effect and evolution of the polarization (**c**) of the semiconductor medium during the SR emission generation. The sample length *L* is 70 μm, and the absorber section length is 20 μm. The product of the small-signal gain *g*_0_ by *L* is 11, *κ*=0.3, *δ*=0.024 and *T*_2_=100 fs. The time step is 0.2 fs.

**Figure 8:**
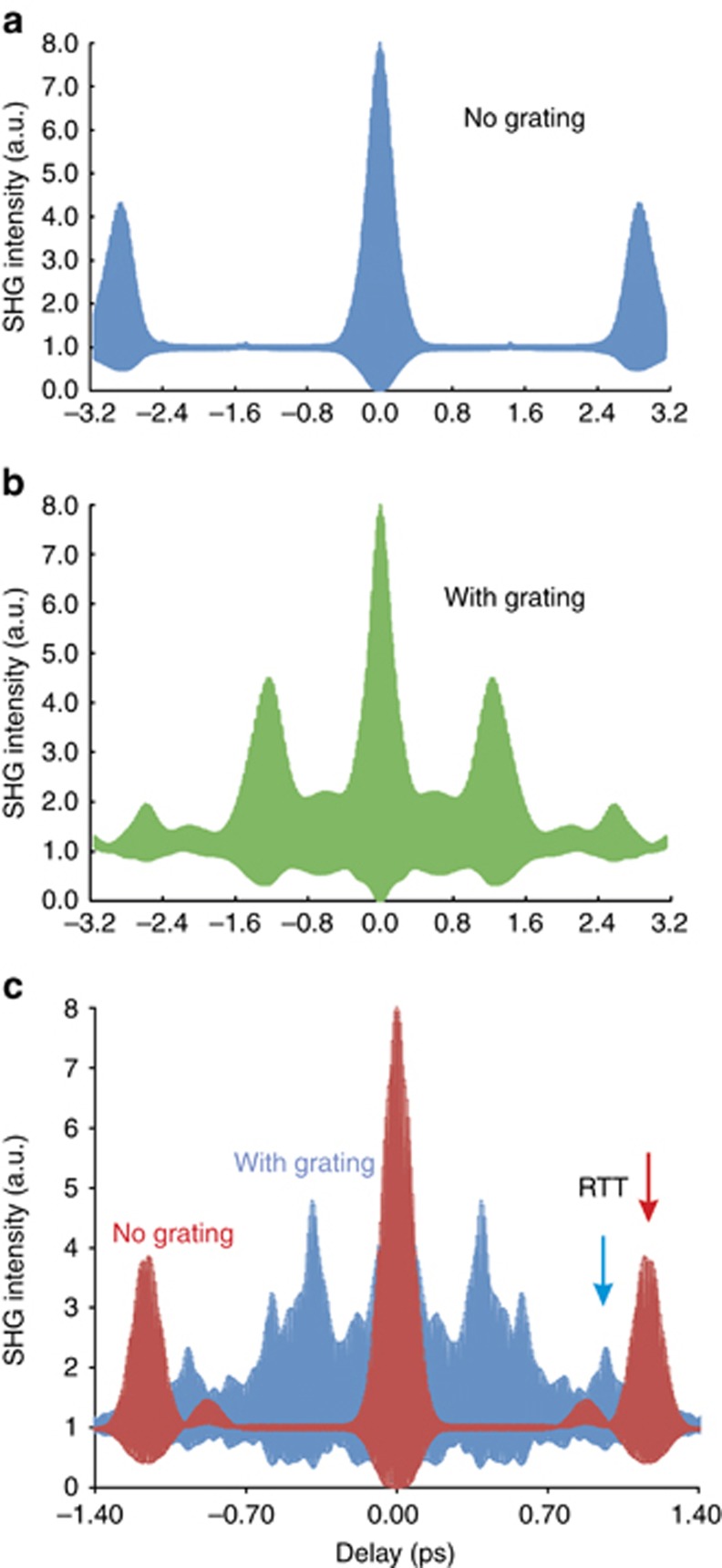
Calculated SHG autocorrelation traces of SR pulses without (**a**) and with (**b**) the grating effect for *δ*=0.024, *κ*=0.35, and *g*_0_*L*=10 and *g*_0_*L*=14 (**c**).

The initial value of *N*(*z*,*0*) is 1 at the end amplifying sections and 0 at the central absorber, and the field reflectivity of the chip facets is 0.57. Due to the random nature of the initialization process of the SR emission, the individual distributions of *N*_1_(*z*,*t*) are also random. The coherence of the interaction of *E*^*±*^ and *N*(*z*,*t*) results in a build-up of negative values of both variables. Ultrafast variations of the carrier density are accompanied by ultrafast oscillations of the induced polarization of the medium *P*^±^(*z*,*t*). Figure 7 shows the output SR pulses (a) and (b) and oscillations of the e–h polarization (c) during the generation of SR pulses with (blue line) and without (red line) the transient grating. The coherent transient grating induced in the medium strongly enhances the polarization oscillations (Figure 7c) and results in the generation of shorter and more powerful SR pulses. These ultrafast oscillations lead to the emergence of additional peaks in the fringe-resolved SHG autocorrelations of SR pulses that are often observed experimentally (see Figure 5)^35, 36^. Figure 8 shows typical fringe-resolved SHG traces of SR pulses generated from a 100-μm-long structure calculated using the traveling wave model, as defined by Equations (1), (2), (3), (4), (5), (6), (7). Figure 8a–8c presents the average of 40 individual SR realizations. The system of equations for the ‘no grating’ case is solved for *δ*=*κ*=*N*_1_=0. Figure 8 clearly shows that the inclusion of the transient grating effect results in the reduction of the RTT of the SR pulses. Without taking the grating into account, the RTT of the sample is 2.9 ps. However, the presence of the grating reduces the RTT value to 2.6 ps. The peaks corresponding to the RTT are shown by arrows in Figure 8. In addition, the transient population gratings are responsible for the additional peaks within the RTT (compare with the experimental results shown in Figure 5). The individual shape of the SHG peaks depends strongly on the small-signal gain *g*_0_, initial value of the carrier density, the polarization relaxation time *T*_2_ and the values of *δ* and *κ*. This model therefore demonstrates the importance of coherent gratings in the SR-generation process in GaAs/AlGaAs semiconductor devices at room temperature.

## Results and discussion

It has previously been established^22, 23, 24, 25^ that SR in semiconductors exhibits a large red shift of the emission wavelength and occurs at much larger e–h concentrations than those in conventional lasing. There are two additional effects due to the substantial nonlinear refractive index change in semiconductor heterostructures. The first is a decrease of the material refractive index with increasing wavelength (the chromatic dispersion). The second effect is a dependence of the refractive index of a semiconductor on the injected carrier density. These effects can contribute to the measured variations of the group refractive index because they both result in a decrease of the refractive index. The impact of these effects can be estimated.

The active layer of all the tested structures is intrinsic bulk GaAs. The chromatic dispersion coefficient is *dn*/*dλ*=−1.156 μm^−1^ in the range of from 800 to 900 nm. A typical red shift of the peak wavelength of SR emission is up to 20 nm with respect to the lasing wavelength^22^, which gives a change of the refractive index of ~−0.6%. Carrier-induced changes in the refractive index of group III–V semiconductors have been intensively studied both experimentally and theoretically (see, for instance, ref. 37 and references therein). Three effects, bandfilling, band-gap shrinkage and free-carrier absorption, may produce sizeable contributions to the total change in refractive index Δ*n*. The typical carrier density at which SR-phase transition takes place in bulk GaAs is ~6 × 10^18^ cm^−3^, whereas the lasing threshold e–h concentration is ~(1.5–2.0) × 10^18 ^cm^−3^ in the same structures^26^. As calculated in ref. 37, this difference of the e–h density can lead to a total change of the refractive index of Δ*n~*−0.01,which is <1%. Therefore, the chromatic dispersion and carrier-induced variations of the refractive index contribute ~1% of the total value of the refractive index change and cannot be the reason for the large decrease of the group refractive index observed experimentally.

The importance of coherent population gratings for the generation of SR emission in semiconductor structures has not been elucidated in previous studies^5, 6, 7, 22^. Here, we note two issues that have been clarified during the present work. First, a sufficiently large value of coupling between oscillators and the radiation field is required for a system to exhibit an SR quantum-phase transition at a certain temperature^12, 13^. In our case of multiple-section semiconductor structures, the coupling is enhanced by (1) the existence of an internal resonator and reflections of the radiation field at the cleaved chip facets and (2) the presence of optical gain at the excitonic part of the spectrum^25^. The emergence of population gratings makes light-matter coupling more efficient and facilitates the SR-phase transition. The grating results in forward–backward coupling of counter-propagating SR pulses, ensuring the establishment of mutual coherence of the electromagnetic field and the e–h system. The reflectivity of the facets of the GaAs/AlGaAs samples under test is much greater than the much lower reflection coefficients of the solid-state and gaseous samples in previous SR studies^3, 4^. Therefore, the effect of forward–backward coupling of counter-propagating SR pulses and transient population gratings in semiconductor structures is much larger.

Coherent population oscillations, which are associated with the transient gratings, result in wave mixing between different spectral modes of counter-propagating SR pulses^20^, which in turn leads to a change in the relative amplitude and phase of the spectral modes and consequently causes variations of the group velocity. A spatial periodic modulation of the e–h density results in both gain/absorption and refractive index modulations, the latter being particularly strong in semiconductor media. Mutual scattering of spectral components of the SR pulses on those spatial gratings is responsible for the observable large changes of the group refractive index.

The second issue is the self-organization from homogeneous into a periodically patterned distribution in the e–h ensemble. Self-organization of emitters has recently been shown to play an essential role in Dicke quantum-phase transition in Bose condensates coupled to a single mode of an optical cavity^14, 38, 39^. Cold-atom trapped Bose condensates experience self-organization and Dicke phase transition when they are pumped transversely by a laser. The characteristic *λ*/2 spatial periodicity has been predicted and experimentally observed. In the SR emission generation described here, phase transition builds up within the optical cavities formed by the cleaved facets. The e–h self-organization and the growth of carrier gratings are caused by two counter-propagating resonant internal electromagnetic fields at the excitonic part of the spectrum, bouncing back and forth between the chip facets. The number of optical modes within a certain bandwidth obviously depends on the sample length. A typical optical bandwidth of the femtosecond SR pulses in our experiments is ~3–4 nm^22, 24, 25^, which means that there are 3–4 or 15–20 modes within this bandwidth for 100 and 450 μm-long samples, respectively. Each individual optical mode forms its own *λ*/2 population sub-grating. When more modes take part in the formation of the cumulative carrier grating, the spatial variations of the e–h density are efficiently averaged out, which inevitably implies that the effect of transient coherent gratings is more pronounced in shorter samples, where just a few optical modes contribute to the cumulative carrier grating. Figure 6 demonstrates that short (100–150 μm long) structures exhibit greater decrease of *n*_*g*_. In addition, coherent beating of the electromagnetic field, optical doublets and triplets is always observed in short samples but never in long (>250 μm) ones ^22, 35, 36^. These facts support the importance of the coherent population gratings in SR generation by semiconductor devices.

## Conclusions

This paper reports an experimental and theoretical study of the role of coherent transient gratings in causing superluminal pulse propagation during SR emission generation in semiconductor heterostructures. The group refractive index can be reduced by ~30–40% during superradiant-phase transition in semiconductors, much larger than the change due to normal laser emission. The superluminal pulse propagation is attributed to the coherent pulse amplification in the semiconductor-active medium and the formation of coherent transient gratings of the population inversion. The observed phenomenon gives definitive evidence that the SR emission generation is fundamentally different from normal lasing, despite many similarities between SR multiple-contact structures and Q-switched semiconductor lasers of similar semiconductor compositions.

The observed substantial decrease of the group refractive index is another feature of the non-equilibrium, macroscopically ordered, BCS-like e–h condensed state formed in the semiconductor during the SR quantum-phase transition^2, 22, 23^. We have reported the importance of coherent population gratings, which have been neglected during previous investigations of SR emission generation in semiconductors. The inclusion of the transient grating effect enables a better understanding of the SR quantum-phase transition and allows a complete physical description.

## References

[bib1] Dicke RH. Coherence in spontaneous radiation processes. Phys Rev 1954; 93: 99–110.

[bib2] Vasil’ev PP. Superradiance: exploiting quantum phase transition in the real world. IEEE Photon Soc News 2014; 28: 4–8.

[bib3] Skribanowitz N, Herman IP, MacGillivray JC, Feld MS. Observation of Dicke superradiance in optically pumped HF gas. Phys Rev Lett 1973; 30: 309–312.

[bib4] Florian R, Schwan LO, Schmid D. Time-resolving experiments on Dicke superfluorescence of O_2_-centers in KCl. Two-color superfluorescence. Phys Rev A 1984; 29: 2709–2715.

[bib5] Scheibner M, Schmidt T, Worschech L, Forchel A, Bacher G et al. Superradiance of quantum dots. Nat Phys 2007; 3: 106–110.

[bib6] Dai DC, Monkman AP. Observation of superfluorescence from a quantum ensemble of coherent excitons in a ZnTe crystal: evidence for spontaneous Bose-Einstein condensation of excitons. Phys Rev B 2011; 84: 115206.

[bib7] Jho YD, Wang X, Reitze DH, Kono J, Belyanin AA et al. Cooperative recombination of electron-hole pairs in semiconductor quantum wells under quantizing magnetic fields. Phys Rev B 2010; 81: 155314.

[bib8] Frolov SV, Gellermann W, Ozaki M, Yoshino K, Vardeny ZV. Cooperative emission in π-conjugated polymer thin films. Phys Rev Lett 1997; 78: 729–732.

[bib9] Andreev AV, Yemel’yanov VI, Il’inskii YA. Cooperative Effects in Optics: Superradiance and Phase Transitions. Bristol: Institute of Physics Publishing. 1993.

[bib10] Hepp K, Lieb EH. On the superradiant phase transition for molecules in a quantized radiation field: the Dicke maser model. Ann Phys 1973; 76: 360–404.

[bib11] Wang YK, Hioe FT. Phase transition in the Dicke model of superradiance. Phys Rev A 1973; 7: 831–836.

[bib12] Carmichael HJ, Gardiner CW, Walls DF. Higher order corrections to the Dicke superradiant phase transition. Phys Letts A 1973; 46: 47–48.

[bib13] Garraway BM. The Dicke model in quantum optics: Dicke model revised. Phil Trans Roy Soc A 2011; 369: 1137–1155. 21320910 10.1098/rsta.2010.0333

[bib14] Baumann K, Guerlin C, Brennecke F, Esslinger T. Dicke quantum phase transition with a superfluid gas in an optical cavity. Nature 2010; 464: 1301–1306. 20428162 10.1038/nature09009

[bib15] Keeling J, Berloff NG. Exciton-polariton condensation. Contemp Phys 2011; 52: 131–151.

[bib16] Schwan LO, Schwendimann P, Sigmund E. Correlations in extended high-density superfluorescence: a self-organized distributed feedback laser. Phys Rev A 1989; 40: 7093–7096. 10.1103/physreva.40.7093 9902122

[bib17] Haake F, Kolobov MI, Steudel H. Dynamical models for forward-backward coupling in superfluorescence. Opt Commun 1992; 92: 385–392.

[bib18] Hau LV, Harris SE, Dutton Z, Behroozi CH. Light speed reduction to 17 metres per second in an ultracold atomic gas. Nature 1999; 397: 594–598.

[bib19] Wang LJ, Kuzmich A, Dogarlu A. Gain-assisted superluminal light propagation. Nature 2000; 406: 277–279. 10917523 10.1038/35018520

[bib20] Bigelow MS, Lepeshkin NN, Boyd RW. Superluminal and slow light propagation in a room-temperature solid. Science 2003; 301: 200–202. 12855803 10.1126/science.1084429

[bib21] Boyd RW, Gauthier DJ. Controlling the velocity of light pulses. Science 2009; 326: 1074–1077. 19965419 10.1126/science.1170885

[bib22] Vasil’ev PP. Femtosecond superradiant emission in inorganic semiconductor. Rep Prog Phys 2009; 72: 076501.

[bib23] Vasil’ev PP, Olle V, Penty RV, White IH. Long-range order in a high-density electron-hole system at room temperature during superradiant phase transition. Europhys Lett 2013; 104: 40003.

[bib24] Vasil’ev PP, Kan H, Ohta H, Hiruma T. Experimental evidence of condensation of electron-hole pairs at room temperature during femtosecond cooperative emission. Phys Rev B 2001; 64: 195209.

[bib25] Vasil’ev PP. Conditions and possible mechanism of condensation of e–h pairs in bulk GaAs at room temperature. Phys Stat Solidi (b) 2004; 241: 1251–1260.

[bib26] Vasil’ev PP. Ultrafast Diode Lasers: Fundamentals and Applications. Norwood: Artech House. 1995.

[bib27] Basov NG, Ambartsumyan RV, Zuev VS, Kryukov PG, Letokhov VS. Nonlinear amplification of light pulses. Sov Phys JETP 1966; 23: 16–22.

[bib28] Kryukov PG, Letokhov VS. Propagation of a light pulse in a resonantly amplifying (absorbing) medium. Sov Phys Uspekhi 1970; 12: 641.

[bib29] Icsevgi A, Lamb WE. Propagation of light pulses in an amplifier. Phys Rev 1969; 185: 517.

[bib30] Lamb GL Jr. Analytical description of ultrashort optical pulse propagation in a resonant medium. Rev Mod Phys 1971; 43: 99–124.

[bib31] Vasil’ev PP, White IH. Gain-enhanced optical coherence in a high optical gain semiconductor. Phys Letts A 2012; 376: 2270–2273.

[bib32] Hopf FA. Phase-wave fluctuations in superfluorescence. Phys Rev A 1979; 20: 2064–2073.

[bib33] Jansen D, Stahl A. Correlation between counterpropagating pulses in superfluorescence. Europhys Lett 1992; 18: 33–38.

[bib34] Jones DJ, Zhang LM, Carroll JE, Marcanac DD. Dynamics of monolithic passively mode-locked semiconductor lasers. IEEE J Quant Electron 1995; 31: 1051–1058.

[bib35] Vasil'ev PP. Role of a high gain of the medium in superradiance generation and in observation of coherent effects in semiconductor lasers. Quant Electron 1999; 29: 842–846.

[bib36] Vasil’ev PP. Superfluorescence in semiconductor lasers. Quant Electron 1997; 27: 860–865.

[bib37] Bennett BR, Soref RA, Del Alamo JA. Carrier-induced change in refractive index of InP, GaAs, and InGaAsP. IEEE J Quant Electron 1990; 26: 113–122.

[bib38] Nagy D, Szirmai G, Domokos P. Self-organization of a Bose-Einstein condensate in an optical cavity. Eur Phys J D 2008; 48: 127–137.

[bib39] Baumann K, Mottl R, Brennecke F, Esslinger T. Exploring symmetry breaking at the Dicke quantum phase transition. Phys Rev Lett 2011; 107: 140402. 22107178 10.1103/PhysRevLett.107.140402

